# Psychometric Properties and Gender Invariance of the Portuguese (European) Version of the Aging Sexual Knowledge and Attitudes Scale

**DOI:** 10.1007/s10823-024-09517-6

**Published:** 2024-09-26

**Authors:** Liliana Rodrigues, Maria Manuela Peixoto, Ana Luísa Patrão, Luís Santos, Sara Isabel Magalhães, Conceição Nogueira

**Affiliations:** 1 Center for Psychology at University of Porto, University of Porto, Porto, Portugal; 2 University Fernando Pessoa, Porto, Portugal; 3 Faculty of Psychology and Educational Sciences at the University of Porto, Porto, Portugal; 4 Institute of Collective Health, Federal University of Bahia, Salvador, Brazil

**Keywords:** Aging, Attitudes, Gender invariance, Knowledge, Psychometric properties, Older people, Sexuality

## Abstract

The sexuality of older people was understood as non-existent or as something outside the prevailing norm. In this sense, analysing people's sexual knowledge and attitudes towards older people is a challenge for theory and practice. The aim of this study is to translate and validate the Aging Sexual Knowledge and Attitudes Scale (ASKAS) for the Portuguese population. A sample of 994 Portuguese adults (70.9% women, n = 705) completed the ASKAS-PT along with a series of self-report measures. Confirmatory factor analysis and the psychometric properties of the Portuguese (European) version of ASKAS-PT were investigated, particularly reliability, temporal stability, and convergent and critical validity. This study also examined the gender measurement invariance of the ASKAS-PT. After confirmatory factor analysis, a two-factor model fit the Portuguese version best. Reliability and validity results also showed good results, and the ASKAS-PT appears to be a gender-invariant measure. Overall, the Portuguese version of the ASKAS showed good psychometric properties and appears to be a valid and reliable measure for assessing knowledge and attitudes about aging.

## Introduction

According to the World Health Organization [WHO] (WHO, 2001), the term "elderly" refers to persons who are 65 years of age or older. The aging process encompasses a developmental phase that has changed over the years as fertility rates have decreased and life expectancy has increased exponentially (Adana et al., 2015). Among the various dimensions that influence quality of life and well-being across the lifespan, sexual health has been described as a relevant dimension (Flynn et al., 2016; Souza Jr., et al., 2021). Thus, sexuality has been described as a relevant aspect of human development and defined by WHO as "*a central aspect of being human throughout the lifespan that encompasses sex, gender identity and role, sexual orientation, eroticism, pleasure, intimacy, and reproduction.* (…) *Sexuality is influenced by the interplay of biological, psychological, social, economic, political, cultural, ethical, legal, historical, religious, and spiritual factors.*" (WHO, 2010, p.10).

Although there is a decline in sexual frequency in late life, it cannot be concluded that there is no longer sexual expression at this stage of development (Lindau et al., 2007). This is consistent with the definition of sexuality that includes affection, caring, and intimacy as relevant dimensions of sexual expression (WHO, 2010). In addition, the ability of family, staff, and caregivers of older adults to address sexual issues can create a safe and secure environment in which physical and emotional intimacy can be expressed and experienced (Langer-Most & Langer, 2010). Lack of knowledge and understanding about sexuality in older adults has been linked to intolerant and conservative attitudes toward sexuality in older adults, particularly in relation to HIV (Aguiar et al., 2012) or dementia (Bauer et al., 2013; Chen, et al., 2017; Jones & Moyle, 2016). Therefore, it is of utmost importance to promote empirical research on knowledge and attitudes about sexuality in older people and to support psychoeducational interventions on this topic.

### Aging Sexual Knowledge and Attitudes Scale

Recognizing the existing gap in the literature, White (1982) developed a self-report instrument consisting of 61 items in 1982 to assess (i) sexual knowledge and (ii) sexual attitudes toward sexuality in older people, the Aging Sexual Knowledge and Attitudes Scale (ASKAS). The ASKAS was originally developed for older people, for people who work with older people on a daily basis, or for people who have a close relationship with older people and are involved in their care and well-being, such as family members or caregivers (White, 1982). The items were developed following the existing literature on sexual changes that occur as part of the aging process and aim to assess sexual knowledge about these changes as well as sexual attitudes toward sexual activities and behaviors in older people (White, 1982).

The ASKAS was the first validated and reliable self-report instrument to assess sexual knowledge and attitudes in older adults and has been used extensively to evaluate the effectiveness of sexual education programs for caregivers in nursing and retirement homes (Baueret al., 2013; Begley et al., 2022; Bouman et al., 2007; Jones & Moyle, 2016; Ng & Boey, 2020). Although the ASKAS is considered a highly relevant self-report questionnaire, it has only been translated and validated for a small number of languages, such as Brazilian (Viana et al., 2010, 2012), Chinese (Yan & Lee, 2013), and Dutch (Mathieu et al., 2013), and consequently most studies have been conducted using the original version in the United States, which introduces a cultural bias to this topic.

### Sexual Knowledge and Attitudes towards Older People and Healthcare Professionals

Educational programs for nursing or residential caregivers should include empirical and valid information about sexuality in later life (Bouman et al., 2007). A review of education and training resources for caregivers who work directly with older people on a daily basis found a lack of resources, highlighting the need for investment in this particular area (Horne et al., 2021). The nursing sector is particularly relevant in this area and has received much attention in recent decades. Nursing students express that they need more information about the sexuality of older people in order to provide them with targeted and timely care (Luketich, 1991). Recent studies have also emphasized the need to adequately address older adults' sexuality in nursing curricula (Wilschut et al., 2021). Research has also found that nursing students who are more knowledgeable about older adults' sexuality have more permissive and tolerant attitudes toward sexuality in older adults (Adana et al., 2015). Data from studies of educational programs on older people's sexuality show that nurses working with older people have more appropriate and permissive attitudes toward sexuality in old age (Akamine et al., 2004; Bauer et al., 2013), including those related to the sexuality of same-sex couples or people with dementia (Bauer et al., 2013). However, contradictory information was found in a very recent study: Nurses who have received formal training on sexuality in the elderly report more knowledge on the topic, but still have conservative attitudes toward sexuality in the elderly (Evangelista et al., 2019). More studies with diverse cultural backgrounds are needed to further explore conservative and permissive attitudes toward sexuality in the elderly, so we have yet to develop empirical and validated self-report measures.

Physicians also complain of limited information on sexuality among older people, despite having permissive and positive attitudes toward sexuality in old age (Dogan et al., 2008). In addition, medical personnel, such as gynecologists, were found to have acceptable knowledge regarding sexuality and aging, but the same was not true for permissive and tolerant attitudes, and non-permissive attitudes toward sexuality in aging correlated with the age of the medical personnel (Langer-Most & Langer, 2010).

In addition, the need for information about knowledge in this area is also paramount for social work students and professionals to promote more permissive attitudes toward sexuality in old age (Gewirtz-Meydan et al., 2017). Considering that more permissive and positive attitudes toward sexuality among older people are associated with more satisfying sex lives (Cybulski et al., 2018), improving quality of life across the lifespan requires, among other dimensions, improving sexual health by promoting knowledge and attitudes about sexuality among older people among health professionals, nurses, social workers, psychologists, and home care workers, especially in diverse cases such as people with dementia (Chen et al., 2017) or lesbian, gay, and bisexual older people (Ahrendt et al., 2017; Willis et al., 2017).

### Study Purpose

Literature reviews emphasize the need for more sexuality education materials for older adults (Chen et al., 2020; Horne et al., 2021). In addition, reliable and validated measures to assess the knowledge of staff working with older people are paramount (Chen et al., 2020) to evaluate current knowledge and attitudes about sexuality among older people and to assess the effectiveness of educational programs. In addition, research studies addressing sexuality in older people emphasize the role of cultural dimensions (Park et al., 2020), suggesting that studies should be conducted with diverse populations that take into account the sociocultural background of the community. Considering the extensive research on knowledge and attitudes about sexuality among older people in the United States and the fact that there is no valid and reliable measure to assess sexual knowledge and attitudes among older people for the Portuguese (European) language, this study aimed to translate and validate the ASKAS for the Portuguese (European) language. This study will examine whether the proposed two-factor structure of the original version of the ASKAS is transferable to the Portuguese population using confirmatory factor analysis, and will evaluate the psychometric properties of the Portuguese (European) version of the ASKAS, specifically reliability (temporal stability and internal consistency) and convergent and critical validity. Finally, this study will examine the measurement invariance of the ASKAS between genders.

## Materials and Methods

### Participants

A total of 994 Portuguese adults (29.1% men, n = 289) participated in the current study. The mean age of the participants was 28.76 (SD = 14.11), ranging from 18 to 86 years. Most of the sample had 12 years of schooling (58.6%, n = 582), were single (73.1%, n = 727), within a relationship (60.8%, n = 604), and lived in an urban area (74.9%, n = 745). Detailed sociodemographic characteristics are presented in Table 1. In addition, a total of 20 Portuguese adults (50% men, n = 10) participated in temporal stability test, and complete the Portuguese version of the ASKAS within a 4-week interval. Half of the sample was single (50%, n = 10), and the majority identified themselves as heterosexual (65%, n = 13).

**Table 1:** Sociodemographic characterization (N = 994)

	n	%
Sex		
* Male*	289	29.1
* Female*	705	70.9
Gender		
* Men*	285	28.7
* Women*	700	70.4
Sexual Orientation		
* Heterosexual*	887	89.2
* Bisexual*	60	6.0
* Homosexual*	36	3.6
* Other*	8	0.8
Educational Level		
* 4 years*	20	2.0
* 6 years*	14	1.4
* 9 years*	32	3.2
* 12 years*	582	58.6
* 13 years or more*	345	34.7
Marital status		
* Single*	727	73.1
* Married/in cohabitation*	214	21.5
* Divorced/separated*	37	3.7
* Widower*	14	1.4
Residence area		
* Urban*	745	74.9
* Rural*	249	25.1
Live with someone older 65y		
* No*	838	84.3
* Yes*	146	14.7

### Procedures

At first, the authors who developed the ASKAS were contacted in order to ask for permission to translate and validate the scale for the Portuguese population. After consent was given by the authors, and all other self-report measures receive authorization to be enrolled in the current study, the project was submitted to the Ethical Committee from the Faculty of Psychology and Educational Sciences at the University of Porto. Once all consents were obtained, recruitment of the sample took place between 2018 and 2020, with recruitment taking place on university campuses in Portugal and in elderly care centres. Public and private universities were invited to enrol in the study, including students and all staff (e.g., professors, researchers, administrative staff, among others), and elderly care centres throughout the country were also approached to participate in the study, with staff (e.g., nurses, occupational therapists, physicians) and family members invited to participate.

Portuguese individuals over the age of 18 were invited to participate in a 15- to 20-min survey. All participants were informed of the voluntary and anonymous nature of the study and were asked to sign an informed consent form if they agreed to participate. The Portuguese (European) version of the ASKAS, other instruments selected for validation purposes, and a sociodemographic sheet were completed by each participant alone and then returned to a researcher, separately from the informed consent form, to ensure anonymity of participation. There was no financial support, compensation, or other incentives for participation in the study. All ethical standards and procedures established by Portuguese legislation and the Helsinki Declaration were followed.

#### Linguistic Validation

The original version of ASKAS was independently translated from English into Portuguese (European) by two researchers, native Portuguese speakers who are proficient in English. The back translation from Portuguese (European) to English was performed by two other researchers and then back translated from Portuguese to English by two researchers, native Portuguese speakers who are proficient in English. The two versions (i.e., the translated and the back-translated) were compared and discussed with another research expert in the field. Inconsistencies were corrected as no semantic differences were found between the English and Portuguese (European) versions of the ASKAS. After completion of the preliminary version, a pilot study was conducted with a group of five men and five women to identify possible problems with the items and to clarify instructions for completing the scale. Since the results of the pilot study did not raise any major concerns and were satisfactory, a final version was produced and no further changes were made to the Portuguese (European) version of the ASKAS.

### Measures

#### Sociodemographic Information

A sociodemographic questionnaire was created for this study to collect personal information. The sociodemographic information collected was age, sex, gender identity, sexual orientation, civil status, education level, residence, and household over 65.

#### Aging Sexual Knowledge and Attitudes Scale (ASKAS)

The ASKAS (White, 1982) is a self-report questionnaire consisting of 61 questions developed to assess the sexual knowledge and attitudes towards older persons. Questions 1 through 35 are designed to assess the sexual knowledge towards older persons. The answers were given according to the criteria of true, false, and do not know with values of 1, 2, and 3, respectively. Questions 36 to 61 are used to assess the sexual attitudes towards older people. Responses were given on a seven-point *Likert* scale (1 = strongly disagree; 4 = neither agree nor disagree; 7 = strongly agree). Scores for each subscale are calculated as the sum of the items. Lower scores on the knowledge subscale indicate high knowledge about sexuality towards the elderly (considering that "do not know" is answered as 3), and higher scores on the attitudes subscale indicate more permissive attitudes toward sexuality among the elderly. The original study suggests good to excellent psychometric properties, including reliability, temporal stability, and validity (White, 1982).

#### Attitudes Toward the Elderly (ATE)

The ATE (Inglehart, et al., 2014) consists of a series of five items from the 2010–2012 World Values Survey that assesses global attitudes toward older people. All questions are answered on a four-point *Likert* scale (1 = strongly disagree; 2 = strongly disagree; 3 = agree; 4 = strongly agree). The score is calculated as the sum of all items, with higher scores expressing less positive attitudes toward older people.

#### Sociosexual Orientation Inventory-Revised (SOI-R)

The SOI-R (Penke & Asendorpf, 2008) is a self-report instrument that measures the main dimensions of sociosexuality: sociosexual behavior, sociosexual attitude, and sociosexual desire. The SOI-R includes 9 items, three items for each subscale, answered on a nine-point Likert scale. Scores for each subscale are calculated by summing the items, with higher scores indicating overt sociosexual orientation. The original study supported the three-factor model and showed that the SOI-R has good to excellent psychometric properties, including reliability and validity (Penke & Asendorpf, 2008). The Portuguese version of the SOI-R also supports the three-factor model and describes good to excellent psychometric properties (Neto, 2016). In the current study, the Cronbach's alpha for the Sociosexual Attitude subscale was 0.74.

#### Sexual Attitudes Scale (SAS)

The SAS is a self-report instrument developed by Hendrick and Hendrick (1987) to assess sexual attitudes. It was later revised and adapted for a brief version (Hendrick et al., 2006). The original and revised versions of the SAS allow for the assessment of four different dimensions, namely permissiveness, birth control, communion, and instrumentality (Hendrick & Hendrick, 1987; Hendrick et al., 2006). The revised and shortened version contains 23 items assessing sexual attitudes. Responses were given on a five-point *Likert* scale (1 = strongly disagree; 3 = neither agree nor disagree; 5 = strongly agree). The total score and subscale scores were calculated by summing the items, with higher scores indicating more permissive sexual attitudes (Hendrick et al., 2006). The original and revised versions have a four-factor structure, and psychometric studies describe good to excellent properties (Hendrick & Hendrick, 1987; Hendrick et al., 2006). The Portuguese version also had a four-factor structure, and good to excellent psychometric properties were obtained (Alferes, 1999). In the present study, the Cronbach alpha values were 0.84 for the total scale and 0.89 for the permissiveness subscale.

#### Data Analysis

A confirmatory Factor Analysis (CFA) with Maximum Likelihood method using the software IBM AMOS version 27.0 was conducted to analyse if the ASKAS two-factor structure model fit to the data. To assess the adjustment quality the following indexes were used: a) X^2^/df (Relative Chi-square) acceptable scores lower than 5.0; b) TLI (Tucker-Lewis Index), CFI (Comparative Fit Index), and GFI (Goodness-of-Fit Index) superior to 0.80; c) RMSEA (Root Mean Square Error of Approximation) inferior to 0.10 (Arbuckle, 2013; Marôco, 2014). The invariance of the factorial model across gender was assessed through a Multigroup Confirmatory Factor Analysis (MCFA). Measurement invariance was analysed by testing configural, metric, and scalar invariance. Statistical procedure for assessing psychometric properties were performed on data analysis software IBM SPSS version 27.0. The construct validity was assessed testing the Pearson Correlation Coefficient between ASKAS and attitudes towards older person, sociosexual orientation attitudes, attitudes towards sexuality and permissiveness attitudes towards sexuality. Construct validity was also assessed through the Average Extracted Variance (AVE; ≥ 0.50) and Composite Reliability (CR; ≥ 0.70; Netemeyer et al., 2003). Internal reliability was assessed through the Cronbach’s Alpha (≥ 0.70) and Omega Coefficient (≥ 0.70; cf. Dunn et al., 2014). Test–retest reliability was assessed through Pearson correlation Coefficient and the Intraclass Correlation Coefficient (≥ 0.75 for continuous scales; cf. Koo & Li, 2016).

## Results

### Preliminary Analyses

The ASKAS did not contain missing values or outliers. There was no evidence of kurtosis (-1.90 to 1.42) or skewness (-1.48 to 1.82) because all values fell within the acceptable range of ± 2 (Gravetter & Wallnau, 2014). Due to the data were considered normally distributed, all the analyses were conducted using parametric statistics.

### Confirmatory Factor Analysis

The ASKAS two-factor model structure was tested through Confirmatory Factor Analysis (CFA) according to the Maximum Likelihood method. Based on the analysis of the goodness of fit indices the two-factor model presented acceptable adjustment fit values: *X*^*2*^/*df* = 5.32, *CFI* = 0.64, *TLI* = 0.63, *GFI* = 0.73, and *RMSEA* = 0.07 [0.06-0.08], *p* < 0.001. Therefore, Modification Indices (MI) were considered according to the Lagrange Multiplier, and errors correlations were suggested for the following pairs of items: item 2—item 4; MI = 150.315; item 3—item 4, MI = 131.90; item 5—item 6, MI = 374,33; item 5—item 7, MI = 88.84; item 6—item 7, MI = 124.42; item 36 – item 41, MI = 153.89; item 42—item 43, MI = 227.38; item 50—item 51, MI = 290.38; item 52—item 53, MI = 375.14. The final model revealed acceptable to satisfactory adjustment fit values: *X*^*2*^*/df* = 3.06, *CFI* = 0.83, *TLI* = 0.83, *GFI* = 0.82, and *RMSEA* = 0.05 [0.04-0.06], *p* < 0.001. Intercorrelation between the two domains was *r* = 0.24, *p* < 0.001. Factor loadings for Aging Sexuality Knowledge subscale ranged between 0.26 and 0.57, and for Aging Sexuality Attitudes subscale ranged between 0.28 and 0.76 (see Table 2).

**Table 2:** Standardized factorial loadings of the ASKAS

Items	Loadings	
*1. Sexual activity in aged persons is often dangerous to their health	.42	
2. Males over the age of 65 typically take longer to attain an erection of their penis than do younger males	.35	
3. Males over the age of 65 usually experience a reduction in intensity of orgasm relative to younger males	.27	
4.The firmness of erection in aged males is often less than of younger persons	.30	
5. The older female (65 + years of age) has reduced vaginal lubrication secretion relative to younger females	.35	
6. The aged female takes longer to achieve adequate vaginal lubrication relative to younger females	.36	
7. The older female may experience painful intercourse due to reduced elasticity of the vagina and reduced vaginal lubrication	.35	
8. Sexuality is a typically a long-life need	.26	
9. Sexual behavior in older people (65 +) increases the risk of heart attack	.28	
*10. Most males over the age of 65 are unable to engage in sexual intercourse	.44	
11. The relatively most sexually active younger people tend to become the relatively most sexually active older people	.36	
12. here is evidence that sexual activity in older persons has beneficial physical effects on the participants	.54	
13. Sexual activity may be psychologically beneficial to older person	.55	
*14. Most older females are sexually unresponsive	.47	
15. The sex urge typically increases with age in males over 65	.38	
16. Prescription drugs may alter a person’s sex drive	.30	
*17. Females after menopause, have a physiologically induced need for sexual activity	.39	
18. Basically, changes with advanced age (65 +) in sexuality involve a slowing of response time rather than a reduction of interest in sex	.50	
19. Older males typically experience a reduced need to ejaculate and hence may maintain an erection of the penis for a longer time than younger males	.38	
*20. Older males and females cannot act as sex partners as both need younger partners for stimulations	.52	
21. The most common determinant of the frequency of sexual activity in older couples is the interest or lack of interest of the husband in a sexual relationship with his wife	.38	
22. Barbiturates, tranquilizers, and alcohol may lower the sexual arousal levels of aged persons and interfere with sexual responsiveness	.46	
23. Sexual disinterest in aged persons may be a reflection of a psychological state of depression	.49	
24. There is a decrease in frequency of sexual activity with older age in males	.42	
25. There is a greater decrease in male sexuality with age than there is in female sexuality	.43	
26. Heavy consumption of cigarettes may diminish sexual desire	.39	
27. An important factor in the maintenance of sexual responsiveness in the aging male is the consistency of sexual activity throughout his life	.49	
28. Fear of the inability to perform sexuality may bring about an inability to perform sexually in older males	.54	
29. The ending of sexual activity in old age is most likely and primarily due to social and psychological causes rather than biological and physical causes	.50	
*30. Excessive masturbation may bring about an early onset of mental confusion and dementia in the aged	.45	
*31. There is an inevitable loss of sexual satisfaction in postmenopausal women	.47	
32. Secondary impotence (nonphisiologically caused) increases in males over the age of 60 relative to younger males	.41	
33. Impotence in aged males may literally be effectively treated and cured in many instances	.52	
34. In the absence of severe physical disability, males and females may maintain sexual interest and activity well into their 80’s and 90’s	.54	
35. Masturbation in older males and females has beneficial effects on the maintenance of sexual responsiveness	.57	
36. Aged people have little interest in sexuality (aged = 65 + years of age)		.29
37. An aged person who shows sexual interest brings disgrace to himself/herself		.53
38. Institutions such as nursing homes ought not to encourage or support sexual activity of any sort in its residents		.60
39. Male and female residents of nursing homes ought to live on separate floors or in separate wings of the nursing home		.51
40. Nursing homes have no obligation to provide adequate privacy for residents who desire to be alone, either by themselves or as a couple		.43
41. As one becomes older (say past 65) interest in sexuality inevitably disappears		.39
42. If a relative of mine, living in a nursing home, was to have a sexual relationship with another resident I would complain to the management		.67
43. If a relative of mine, living in a nursing home, was to have a sexual relationship with another resident I would move my relative from this institution		.71
*44. If a relative of mine, living in a nursing home, was to have a sexual relationship with another resident I would stay out of it as it is not my concern		.49
45. If I knew that a particular nursing home permitted and supported sexual activity in residents who desired such, I would not place a relative in that nursing home		.62
46. It is immoral for older persons to engage in recreational sex		.60
*47. I would like to know more about the changes in sexual functioning in older years		.36
*48. I feel I know all I need know about sexuality in the aged		.37
*49. I would complain to the management if I knew of sexual activity between any residents of a nursing home		.76
*50. I would support sex education courses for aged residents of nursing homes		.56
*51. would support sex education courses for the staff of nursing homes		.60
*52. Masturbation is an acceptable sexual activity for older males		.68
*53. Masturbation is an acceptable sexual activity for older females		.70
*54. Institutions such as nursing homes, ought to provide large enough beds for couples who desired such to sleep together		.66
*55. Staff of nursing homes ought to be trained or educated with regard to sexuality in the aged and/or disabled		.71
*56. Residents of nursing homes ought to engage in sexual activity of any sort		.60
57. Institutions such as nursing homes should provide opportunities for the social interaction of men and women		.63
58. Masturbation is harmful and ought to be avoided		.61
59. Institutions such as nursing homes should provide privacy so as to allow residents to engage in sexual behavior without fear of intrusion or observation		.76
60. If family members object to a widowed relative engaging in sexual relations with another resident of a nursing home, it is the obligation of the management and staff to make certain that such sexual activity is prevented		.30
61. Sexual relations outside the context of marriage are always wrong		.28

*Items scored reverse; *ASKAS* Aging Sexual Knowledge and Attitudes Scale

### Reliability

The Cronbach’s alpha value for the Aging Sexuality Knowledge subscale of 0.89 and for the Aging Sexuality Attitudes subscale of 0.78 indicated that both subscales had good internal consistency. No changes were observed in Cronbach’s alpha value if items were deleted. The intraclass correlation coefficient estimate and the corresponding 95% confidence intervals were also calculated using the two-way mixed effects model with a single measure (ICC), with results indicated good values for the Aging Sexuality Knowledge subscale (ICC = 0.89; 95% CI [0.88, 0.90]) and for the Aging Sexuality Attitudes subscale (ICC = 0.80; 95% CI [0.78, 0.82]), and the item-total coefficients suggested that the deletion of any of the items would not significantly improve the reliability of both subscales. Coefficient’s omega also indicated that both subscales had good composite reliability, with Coefficient omega value for the Aging Sexuality Knowledge subscale of 0.89 and for the Aging Sexuality Attitudes subscale of 0.82.

Twenty participants were retested 4 weeks after the initial testing to establish the test–retest reliability of the ASKAS scale. Results showed test–retest correlation for the ASKAS Knowledge and the ASKAS Attitudes score were positive and significant and demonstrated good test–retest reliability (Knowledge, *r* = 0.80; *p* < 001; Attitudes, *r* = 0.75, *p* < 0.001). The intraclass correlation coefficient estimate and the corresponding 95% confidence intervals were also calculated using the two-way mixed effects model with a single measure (ICC). Results indicated that the level of absolute agreement between the two-time waves was good (Knowledge, ICC = 0.89; 95% CI [0.70, 0.95]; Attitudes, ICC = 0.74; 95% CI [0.34, 0.90]).

### Convergent and Criterion Validity

To assess the convergent and criterion validity of the ASKAS, Pearson coefficient correlations between the Aging Sexuality Knowledge and Aging Sexuality Attitudes subscales and attitudes towards elderly, sociosexual orientation attitudes, attitudes towards sexuality were performed. As shown in Table 3, the Aging Sexuality Knowledge subscale was positively and significantly associated with attitudes towards elderly (lack of knowledge was associated with negative attitudes towards elderly). The Aging Sexuality Attitudes subscale was positively and significantly associated with attitudes towards elderly (permissive attitudes towards elderly sexuality were associated with positive attitudes towards elderly), and negatively and significantly associated with attitudes towards sexuality in general (permissive attitudes towards elderly sexuality were associated with permissive attitudes towards sexuality in general), with permissive attitudes towards sexuality (permissive attitudes towards elderly sexuality were associated with permissive attitudes towards sexuality in general), and with sociosexual orientation attitudes (permissive attitudes towards elderly sexuality were associated with permissive attitudes towards sociosexual orientation).

**Table 3:** Pearson’s correlations between ASKAS knowledge and ASKAS attitudes, ATE, SOI-R-A, SAS, and SAS-P

	1	2	3	4	5	6
1. ASKAS—Knowledge	1					
2. ASKAS—Attitudes	.24***	1				
3. Attitudes towards elderly	.09*	.32***	1			
4. Sociosexual orientation attitudes	.05	-.19***	.09**	1		
5. Attitudes towards sexuality	-.04	-.20***	.11**	.63**	1	
6. Permissiveness attitudes towards sexuality	.05	-.16***	.15***	.76***	.85***	1

**p < .05 *** *p* < .001

*ASKAS* Aging Sexual Knowledge and Attitudes Scale; *ATE* Attitudes towards Elderly; *SOI-R-A* Sociosexual Orientation Revises—Attitude; *SAS* Sexuality Attitudes Scale; *SAS-P* Sexuality Attitudes Scale—Permissiveness

The Average Variance Extracted (AVE) and the Composite Reliability (CR) were estimated to better explore the construct validity. For the Aging Sexuality Knowledge subscale, the AVE was unsatisfactory (0.29; ≤ 0.50 cf. Netemeyer et al., 2003) and the CR was above the recommended values (0.90; ≥ 0.70; cf. Netemeyer et al., 2003). Likewise, for the Aging Sexuality Attitudes subscale, the AVE was threshold (0.46; ≤ 0.50 cf. Netemeyer et al., 2003) and the CR was above the recommended values (0.93; ≥ 0.70; cf. Netemeyer et al., 2003).

### Invariance of the Factor Structure Between Samples

ASKAS measurement invariance was tested across gender (Table 4). The two-factor model showed a good adjustment to male and female individuals simultaneously (*X*^*2*^*/df* = 1.02; *RMSEA* = 0.03; *CFI* = 0.81; *TLI* = 0.81), revealing configural invariance. The ∆X^2^ and the ∆CFI revealed metric (∆*X*^*2*^(3) = 6.36, *p* = 0.434; ∆CFI < 0.01) and scalar invariance (∆*X*^*2*^(2) = 6.25, *p* = 0.324; ∆CFI = 0.01). These overall results corroborate the presence of measurement invariance across sample.

**Table 4:** Tests of measurement invariance of the ASKAS

		X^2^	df	RMSEA	CFI	TLI	∆CFI
Gender	Configural invariance	60.14	59	.034 [.033-.035]	.81	.81	__
	Metric invariance	66.60	62	.034 [.033-.035]	.81	.81	< .01
	Scalar invariance	66.39	61	.034 [.033-.035]	.80	.80	.01

*ASKAS* Aging Sexual Knowledge and Attitudes Scale; *χ2* Chi-Square; *df* degrees of freedom; *CFI* Comparative Fit Index; *TLI* Tucker-Lewis Index; *RMSEA* Root Mean Square Error of Approximation

## Discussion

The ASKAS was developed to address two distinct domains of sexuality: knowledge of changes (or not) in sexual response and behavior in the elderly and attitudes toward sexual behavior and sexual behavior in older age (White, 1982). As average life expectancy increases, a deeper understanding of knowledge and attitudes about sexuality in older people is of paramount importance to promote quality of life, well-being, global health, and sexual health. Main findings of the current study suggest that the Portuguese version of the ASKAS is a reliable and valid self-report instrument that is gender-invariant and its use is recommended for researchers and caregivers of older people in Portuguese samples to assess knowledge about and attitudes toward sexuality in old age.

A two-factor model was found to replicate the structure proposed by White (1982), with one factor assessing knowledge about changes in sexual behavior and response in older people, and another factor measuring attitudes toward sexual behavior and sexual activity in older people. The goodness-of-fit indices showed good to satisfactory scores, with the RMSEA score indicating adequate and good fit (Xia & Yang, 2019) and CFI, TLI, and GFI suggesting thresholds between 0.82 and 0.83; however, given the sample size and no suggested item deletion, values can be interpreted as adequate (Hooper et al., 2008). Factor loadings for the knowledge about sexuality in old age and attitudes toward sexuality in old age subscales were relatively low in the current study, which was also found in the original scale, particularly for the knowledge subscale (White, 1982), as well as in the Brazilian version (Viana et al., 2012) and the Dutch version (Mahieu et al., 2013). For the original scale, factor loadings for the knowledge about sexuality subscale ranged from 0.29 to 0.61 at older ages and for the attitudes toward sexuality subscale ranged from 0.22 to 0.76 at older ages (White, 1982). One possible explanation is that both the Knowledge and Attitudes subscales may aggregate more specific dimensions.

Reliability results from the current study indicate that ASKAS-PT has good internal consistency as determined by Cronbach's alpha (values of 0.89 and 0.78 for the Knowledge subscale and the Attitudes subscale, respectively), replicating results from the original study (values of 0.90 to 0.93 for the Knowledge subscale and from 0.76 to 0.87 for the Attitudes subscale; White, 1982). The good reliability scores were confirmed by Cronbach Alpha and McDonald Omega (Green & Yang, 2015; Peters, 2014), and both scores were above the threshold of 0.70 (Dunn et al., 2014). Regarding temporal stability, ASKAS-PT also showed reasonable to good temporal stability within a 4-week interval, with Pearson correlation coefficient values above the threshold for continuous scales of 0.75 (Koo & Li, 2016), confirming the original results (White, 1982).

As expected, the convergent and critical validity of the ASKAS-PT showed that individuals who lacked knowledge about sexuality in late adulthood were those who had more negative attitudes toward older people in general (Yan & Lee, 2013). In addition, individuals who reported more permissive attitudes toward sexuality in late life also reported more positive attitudes toward older people in general (Yan & Lee, 2013), more permissive attitudes toward sexuality in general, and more permissive attitudes toward lesbian, gay, and bisexual individuals and, consequently, sociosexual orientation (Willis et al., 2017). In addition, ageist sexual stereotypes have been associated with poorer sexual health later in life (Syme & Cohn, 2021).

The Portuguese version of the ASKAS showed gender measurement invariance, indicating that the two-factor model proposed by the original version (White, 1982) has a good fit in both female and male samples. Although this dimension has not yet been investigated in any study validating the ASKAS, the current results indicate that the Portuguese version of the ASKAS has configural, metric, and scalar invariance across genders, implying that female and male responses to the questionnaire are equivalent and comparable. This new finding will allow gender comparison studies to be conducted with the ASKAS-PT.

Despite the relevance of the current study, some limitations should be considered. First, an online sample survey was conducted, so it is possible that the sample includes participants who are comfortable with Web surveys and that only individuals with Internet access can participate in the study. Nonetheless, online sampling for sexuality-related studies may promote a safer and more anonymous environment. In addition, women are overrepresented in the sample, which could be explained by the overrepresentation of women in sexuality-related research studies conducted in Portugal (de Oliveira et al., 2014; Pascoal et al., 2013). The majority of the sample has an education of 12 years or more, which may have an impact on the mean level of knowledge about sexuality among older people. On the other hand, the majority of participants did not live with an older person, which may also affect knowledge levels and attitudes.

Sexual health and sexuality-related dimensions are important areas for global health and quality of life (Flynn et al., 2016; Souza Jr. et al., 2021). Therefore, the study of sexuality in older age is of utmost relevance, and there is a lack of reliable and valid measurement tools translated and adapted especially for the Portuguese population. The present study aimed to fill this gap in the literature, and the main results provided empirical evidence in support of the Portuguese version of the ASKAS as a reliable and valid self-report measure to assess knowledge about sexual behavior and sexual changes in late life, as well as attitudes toward sexuality in older people. Therefore, the use of ASKAS-PT is recommended for academic and professional purposes to advance scientific and professional knowledge on this topic and to improve caregivers' practices toward older people.

## Data Availability

The datasets generated during and/or analysed during the current study are not publicly available due to ongoing studies but are available from the corresponding author on reasonable request.
